# Immune and molecular landscape behind non-response to Mycophenolate Mofetil and Azathioprine in lupus nephritis therapy

**DOI:** 10.21203/rs.3.rs-3783877/v1

**Published:** 2024-01-12

**Authors:** Raúl López-Domínguez, Juan Antonio Villatoro-García, Concepción Marañón, Daniel Goldman, Michelle Petri, Pedro Carmona-Sáez, Marta Alarcón-Riquelme, Daniel Toro-Dominguez

**Affiliations:** GENYO. Centre for Genomics and Oncological Research: Pfizer, University of Granada, Andalusian Regional Government, PTS Granada; GENYO. Centre for Genomics and Oncological Research: Pfizer, University of Granada, Andalusian Regional Government, PTS Granada; Department of Medical Genomics, Center for Genomics and Oncological Research (GENYO); Johns Hopkins University; Johns Hopkins University; Department of Bioinformatics, Center for Genomics and Oncological Research (GENYO); Department of Medical Genomics, Center for Genomics and Oncological Research (GENYO); GENYO. Centre for Genomics and Oncological Research: Pfizer, University of Granada, Andalusian Regional Government, PTS Granada

## Abstract

Lupus nephritis (LN) represents one of the most severe complications of systemic lupus erythematosus, leading to end-stage kidney disease in worst cases. Current first-line therapies for LN, including mycophenolate mofetil (MMF) and azathioprine (AZA), fail to induce long-term remission in 60–70% of the patients, evidencing the urgent need to delve into the molecular knowledge-gap behind the non-response to these therapies. A longitudinal cohort of treated LN patients including clinical, cellular and transcriptomic data, was analyzed. Gene-expression signatures behind non-response to different drugs were revealed by differential expression analysis. Drug-specific non-response mechanisms and cell proportion differences were identified. Blood cell subsets mediating non-response were described using single-cell RNASeq data. We show that AZA and MMF non-response implicates different cells and regulatory functions. Mechanistic models were used to suggest add-on therapies to improve their current performance. Our results provide new insights into the molecular mechanisms associated with treatment failures in LN.

Systemic Lupus Erythematosus (SLE) is a heterogeneous autoimmune disease with a wide range of severe clinical manifestations. Lupus nephritis (LN) represents one of the most severe complications affecting up to 50% of patients and can lead to end-stage kidney disease, being an independent risk factor for mortality^1,2^. LN is a clinically silent disease mostly detected when irreversible kidney damage is already installed, so effective treatment on time is crucial to stop further progression of the disease.

Immunosuppressant drugs including mycophenolate mofetil (MMF) and azathioprine (AZA) are widely used as induction and/or maintenance therapies for LN, along with initial high-doses of standard of care drugs (SOC), including glucocorticoids (GC) and hydroxychloroquine (HC). Belimumab and calcineurin inhibitors are also prescribed for LN. However, the efficacy of this therapy varies enormously between patients, and 60–70% of LN patients have not reached a long-term remission and a complete renal response one year after the treatment^3,4^. Additionally, chronic exposure to SOC leads to serious side effects due to drug-induced toxicity^5^, although immunosuppressive drugs potentially enhance renal recovery and facilitate quick tapering of corticosteroids^3,4^. Therefore, there is an urgent need to delve into the molecular knowledge-gap behind the non-response to these drugs with the goal of reducing therapeutic failure and improving long-term prognosis.

Treat-to-target approaches in which personalized molecular patterns guide therapeutic decisions are rapidly growing in the medical field, primarily in oncology^6,7^, but remain largely unmet in clinical rheumatology^8^. In this context, some gene variants have been proposed to be used to adjust AZA doses in individual patients^9^ while inosine monophosphate dehydrogenase activity has been used as biomarker of MMF efficacy following organ transplantation^10^. In this regard, mycophenolic acid (MPA) levels in blood have been correlated with disease state and with the appearance of flares, being associated with persistent remission rates for concentrations higher than 3.5 mg/L. It has also been observed that even if MMF doses are increased, the concentration of MPA does not always increase, with no direct correlation between the two^11^. Therefore, individual differences should always be considered, including race, age, body weight or even individual cellular or molecular patterns for a potentially more personalized therapeutic dosing^12^.

Omics-based personalized approaches offer a major promise towards high-definition medicine, allowing to dissect the heterogeneity behind the disease, defining new generation biomarkers to tailored treatment strategies^13–16^. Molecular dysregulation in SLE fluctuates with a non-linear clinical course and unpredictable patterns of flares, hindering the development of effective and robust predictive biomarkers for both diagnosis and drug responsiveness in cross-sectional cohorts^17^.

In the present study, a longitudinal cohort of responder and non-responder patients to LN drugs was retrospectively analyzed in order to fill the knowledge-gap behind non-response mechanisms combining transcriptomic, cellular and clinical frameworks. Our results can provide support to a future personalized medicine that is increasingly closer. The possibility to anticipate therapy failures to help to refine the first-line choice of treatment for LN patients can be decisive in reducing the progression of nephritis and the consequent chronic kidney damage.

## Results

### Patients and clinical information

**Figure 1:**
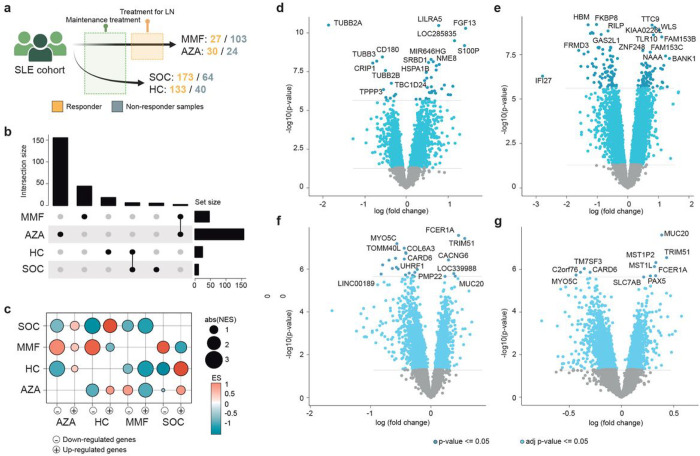
Gene-signatures behind response and non-response to LN therapies. **a**, Therapeutic scheme followed for the patients. **b**, barplots show the number of DEGs for each drug (set size) and number of shared genes between drugs (intersection size). **c**, GSEA scores obtained comparing up and down-expressed gene sets for each drug (columns) with the full lists of genes ranked by fold-change for the rest of drugs (rows). ES: enrichment score; NES: normalized enrichment score. **d**, **e**, **f**, **g**, volcano plot distribution of p-values and fold-changes for genes comparing responder and non-responder samples for MMF (d), AZA (e), HC (f) and SOC (g), respectively.

Gene expression, serological, demographic and clinical information were longitudinally collected for responder and non-responder patients to MMF, AZA, HC and SOC (HC and HC + GC). The treatment scheme followed is summarized in Fig. 1a. The number of patients and samples for each group along with patient characteristics are presented in Table 1 and expanded in Supplementary Table 1. No differences were found in age and sex in both groups, but non-responders to MMF showed a significantly higher disease activity and an enrichment in African-American ancestry. Higher doses of MMF, prednisone and acetylsalicylic acid (ASA) were observed in non-responders to MMF increased by standard medical decisions in the face of ineffective response to lower doses. Responders to HC and SOC showed an enrichment in non-steroid anti-inflammatory drugs (NSAID) usage. The serological profiles showed differences in C3 and C4 levels, previously associated to renal damage^18^, and anti-dsDNA titers for all drugs (Table 1). Interestingly, anti-dsDNA titers were increased in non-responders, except for MMF non-responders, who showed increases in anticardiolipin IgA antibodies. Regarding disease activity-related clinical components, a significantly higher incidence of SLEDAI proteinuria and other renal manifestations were observed in non-responders considering all visits^19^ (Supplementary Table 1).

### Molecular signatures behind non-response to LN drugs

Initially, lists of differentially expressed genes (DEG) between responder and non-responder samples to each immunosuppressant drug were compared using the Systemic Lupus Erythematosus Responder Index (SRI-4) and the protein/creatinine ratio in urine as response measurements by gene set enrichment analysis (GSEA)^20^. These two response measurements gave highly significant signatures between responder/non-responder groups of patients, and both signatures were similar when using either measurement (enrichment score (ES) = 0.93 and p-value = 4.39e-11 for up-expressed genes and ES = −0.94 and p-value = 5.31e-9 for down expressed genes) (Supplementary Fig. 1a). SRI-4 was used henceforth due to greater data availability. A total of 46, 157, 24 and 11 DEGs between responder and non-responder samples to MMF, AZA, HC and SOC, respectively, with a Bonferroni-corrected p-value < 0.05 were obtained (Fig. 1b). DEG for HC and SOC were extensively shared (Fig. 1b), while up and down-regulated DEG for MMF were down and up-regulated for AZA, respectively, suggesting opposite gene-expression patterns between non-responders to these two medications (Fig. 1c). Only 2 genes were found significant differentiating response and non-response for both drugs, *CLEC4C* and *C15orf54* (Fig. 1b), but in opposite directions.

*CRIP1*, *CD180* and several tubulin-related genes, and on the other hand, *LILRA5*, *NME8* or *S100P* were the genes most up and down regulated, respectively, in non-responders to MMF (Fig. 1d and Supplementary Fig. 1b). The ratio between mean expressions of up and down regulated genes significantly differentiated responder and non-responder patients to MMF, being these expressed in the opposite direction to the gene expression in patients responder or non-responder to AZA, SOC and HC (Supplementary Fig. 1c), suggesting that the gene-signature is exclusively associated with MMF treatment. For AZA, we found genes *BANK1* or TLR10 are most down-regulated, and some interferon type I (IFN-I) regulated genes are up-regulated in non-responders (Fig. 1e and Supplementary Fig. 2a). Most of DEGs for SOC and HC were shared (Fig. 1f–g. and Supplementary Fig. 3a-b), mainly because patients with SOC are treated with GC in combination to HC, highlighting *TRIM51* or *MUC20* in responders. Expression ratios for AZA DEGs significantly and specifically distinguished responders from non-responders to AZA, not to other drugs (Supplementary Fig. 2b), and similar conclusions were obtained for SOC and HC (Supplementary Fig. 3c-d).

Top10 DEGs based on adjusted p-value were used as features to build machine learning (ML) based models with nested 10-fold cross validation to predict response to each drug. As described in Supplementary Fig. 4a, we obtained Matthews Correlation Coefficient (MCC) of 0.7, 0.81, 0.63 and 0.56 for MMF, AZA, HC and SOC (Supplementary Fig. 4b), respectively. Thus, these gene-signatures accurately predicted drug response to each drug, but better for AZA and MMF.

The functionality of DEG was investigated by the quantitative set analysis for gene expression modular analysis (QuSAGE). This analysis revealed over-regulation of B cell and dendritic cell (DC)-related processes, and an under-regulation of NK, CD4^+^ T cells and IFN-I signaling in non-responder patients to MMF. IFN-I and DC-related functions were over-represented in non-responders to AZA, while B cell and T cell activation and differentiation were under-represented for this drug. For SOC and HC, B cell functions were down-regulated in non-responders, and more general biological processes, like cell division and regulation of immune signaling were up-regulated (Supplementary Table 2). So, DEGs for each drug revealed differences in the immune processes occurring in different cell populations.

### Cellular profile influence on response rates

**Figure 2:**
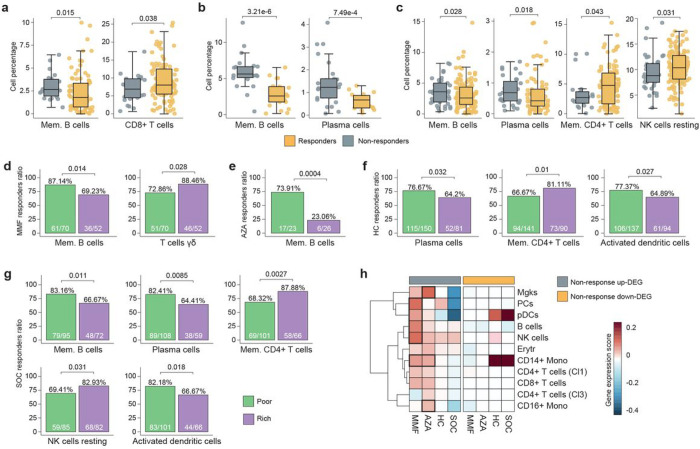
Cell profiles differentiate response rates to LN drugs. **a, b, c**, *In silico* cell percentages significantly different between responder and non-responder patients to MMF, AZA and HC. **d**, **e**, **f**, **g**, Percentage of responders (response rate) based on specific cell type abundance for MMF, AZA, HC and SOC. P-values were calculated by Fisher’s exact test. **h**, Maximum gene-expression scores from means obtained at subcluster level for each major cell population. Gene-expression scores were calculated for each cell using AddModuleScore function from Seurat R package. Up and down-DEG lists from each drug were used as input. The wider borders are used to highlight the higher scores.

*In silico* deconvolution of bulk transcriptomic data was performed to obtain the proportions of 20 different blood cell types in the samples, showing significantly lower CD8^+^ T cell and higher memory B cell proportions in non-responder patients to MMF (Fig. 2a), in line with the previous functional analysis on DEGs obtained. Memory B cells and plasma cells (PC) were increased in AZA (Fig. 2b) and HC non-responder patients, in addition to a decrease in CD4^+^ T cells and NK cells for non-responders to HC (Fig. 2c). Next, samples were stratified based on their cell proportions (see Methods). Certain cell proportions contributed significantly to response to each drug. Significantly higher proportions of responders were associated with poor numbers of memory B cells, PCs and DCs, while the greater the proportion of T and NK cells, the greater the response ratios (Fig. 2d–g).

To further dissect blood cell types and their influence on the response to each drug, public single-cell RNA-seq data from PBMC of 41 SLE patients was analyzed. First, cells were clustered and the major blood cell types were identified (Supplementary Fig. 5a-b). Second, clustering rounds were performed for each major cell type. Using the AddModuleScore function from the Seurat R package^21^, maximum gene-expression scores for up and down-DEG were calculated across subclusters within each major cell type for each drug, in order to identify major cells contributors to the non-response (Fig. 2h). Interestingly, the non-response up-regulated DEGs (up-DEG) for MMF and AZA were expressed in different cell subsets. This suggests that different cell subsets are involved in non-response to each drug. For MMF, non-response up-DEGs were mainly expressed in PCs, B cells, NK cells, plasmacytoid dendritic cells (pDCs) and CD14^+^ monocytes, either for all cells or for some subclusters of cells within them. For AZA, megakaryocytes, CD14^+^ and CD16^+^ monocytes showed the highest scores. On the other hand, non-response up-DEG for HC and SOC were not primarily expressed by any specific cell type, while only pDCs and CD14^+^ monocytes were expressing the genes up-regulated in responders.

### Cell subpopulations behind non-response to LN drugs at single-cell level

**Figure 3:**
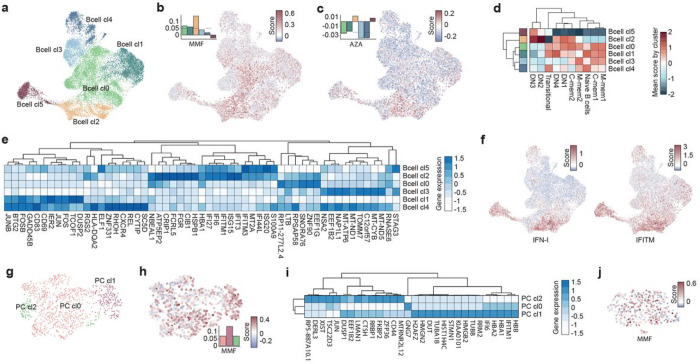
Non-response signature expression across B cell, PC and pDCs subpopulations. **a**, UMAP representation of B cell clusters. **b**, **c**, Gene-expression scores by cells for MMF, and AZA non-response signatures in B cells, respectively. Bar plots show mean scores by cluster. **d**, Mean of gene-expression scores by cluster for different gene-lists of B cell subpopulation markers. **e**, Top 10 most differentially expressed genes between B cell clusters. Color represents expression magnitude. **f**, Gene-expression scores by cells for IFN-I and IFITM-related genes across B cells. **g**, UMAP representation of PC cell clusters. **h**, Gene-expression scores by cells for MMF non-response signature in PCs. Bar plots show mean scores by cluster. **i**, Top 10 most differentially expressed genes between PC clusters. **j**, Gene-expression scores by cells for MMF non-response signature in pDCs. Gene-expression scores were calculated for each cell using AddModuleScore function from Seurat R package. Up and down-DEG lists from each drug were used as input.

Now, clusters associated to each major cell type were subdivided to increase granularity. B cells were divided into 6 clusters (Fig. 3a). The non-response signature for MMF and to a lesser extent for AZA, was mainly expressed by the Bcell_cl2 (Fig. 3b–c). Bcell_cl2 was identified as a cluster of cells phenotypically similar to age-associated B cells (ABCs, also called DN2 cells) (Fig. 3d), characterized by the expression of *CXCR3*, *ITGAX* and *TBX21*. The top-10 DEGs between clusters are shown in Fig. 3e. Bcell_cl2 together with Bcell_cl5 (with a DN3 phenotype) over-expressed IFN-I stimulated genes (ISG) such as *IFIT3*, *IFI27* and *IFITM* (Fig. 3e–f). Of the 3 clusters of PCs (Fig. 3g), the non-response signature to MMF was expressed in all, but more in PC_cl1 (Fig. 3h), which in turn showed greater *IFITM* and *ISG* expression scores (Fig. 3i). In the case of pDCs, most cells expressed the MMF-non response signature (Fig. 3j).

**Figure 4:**
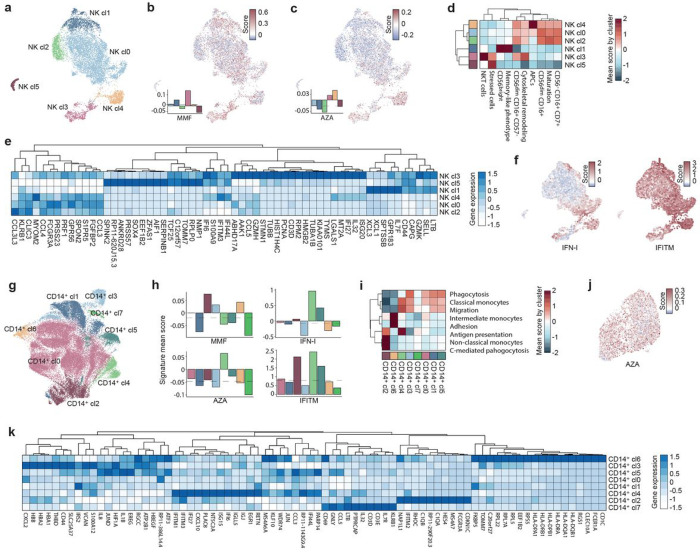
Non-response signature expression across NK cells and monocytes. **a**, UMAP representation of NK cell clusters. **b**, **c**, Gene-expression scores by cell for MMF, and AZA non-response signatures in NK cells, respectively. Bar plots show mean scores by cluster. **d**, Mean of gene-expression scores by cluster for different gene-lists of NK cell subpopulations and biological function-related markers. **e**, Top 10 most differentially expressed genes between NK cell clusters. Color represents expression magnitude. **f**, Gene-expression scores by cells for IFN-I and IFITM-related genes in NK cells. **g**, UMAP representation of CD14^+^ monocytes clusters. **h**, Bar plots show mean gene-expression scores by cluster for MMF and AZA non-response signatures, IFN-I and IFITM-related genes. **i**, Means of gene-expression scores by cluster for different gene-list of monocyte subpopulation and biological functions-related markers. **j**, Gene-expression scores by cells for AZA non-response signature in CD16^+^ monocytes. **k**, Top 10 most differentially expressed genes between CD14^+^ monocyte clusters. Color represents expression magnitude. Gene-expression scores were calculated for each cell using AddModuleScore function from Seurat R package. Up and down-DEG lists from each drug were used as input.

Regarding NK cells, 6 clusters were obtained (Fig. 4a). MMF non-response signature was over-represented in cluster NK_cl3, while AZA non-response signature was mainly expressed in NK_cl4 (Fig. 4b–c). Expression scores for cell and functional markers allowed to annotate the NK_cl3 as CD3 + NKT cells, and NK_cl4 as CD16^+^CD56^−^ NK cells with antigen-presenting (APC)-related functions (Fig. 4d). Of note, both clusters showed high-*IFITM* and *ISG* signatures (Fig. 4e–f).

Additionally, big differences between MMF and AZA signatures was observed in the myeloid compartment. CD14^+^ cells were divided into 8 clusters (Fig. 4g). A high-MMF non-response signature was observed in CD14^+^_cl2 and CD14^+^_cl6 (Fig. 4h). CD14_cl6 showed a high score for adhesion functions and intermediate monocyte phenotypes (Fig. 4i). Since these cells strongly express CD1C, *CLEC10A* and class I *HLA* genes, they likely contain type 2 conventional dendritic cells (cDC2) (Fig. 4k). CD14^+^_cl2 reflected a CD16 + non-classical monocyte phenotype and complement-mediated phagocytosis (Fig. 4i), expressing complement proteins such as *C1QA* and *C1QB* (Fig. 4k). Functionally, these cells are ready to adhere and migrate to the kidney tissue to get differentiated to macrophages and to interact with immune-complexes^22^. An independent and quite large cluster of CD16^+^ monocytes was defined (Supplementary Fig. 5a-b), showing the exclusive and importantly increased expression of the AZA non-response signature (Fig. 4j). AZA non-response signature was also expressed in CD14^+^_cl4, showing antigen presentation and migration functions (Fig. 4h–i). Differences regarding IFN were also found. AZA non-response-related monocyte clusters showed high-*IFITM* and *ISG* genes, but only high-*IFITM* gene expression was observed for clusters expressing the MMF non-response signature (Fig. 4h). The same occurred for CD8^+^ T clusters, although the MMF non-response score in CD8^+^ T cells was weaker (Supplementary Fig. 6a-c). The AZA non-response signature was also highly expressed in a non-IFN related subcluster of megakaryocytes (Supplementary Fig. 6d-g). Thus, we showed that clusters expressing MMF and AZA non-response signatures co-expressed *ISG* and *IFITM* gene signatures (Supplementary Fig. 7).

Finally, the HC and SOC non-response signatures were not particularly expressed in any specific subclusters. Instead, the expression scores were distributed across cells from all subclusters. On the other hand, non-response up-regulated genes for HC and SOC were highly expressed in cDC2 and in pDCs (Supplementary Fig. 6h-k)

### Druggability of regulatory networks of cells influencing non-response

**Figure 5:**
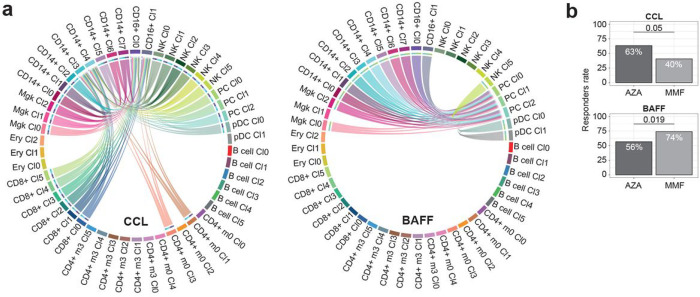
Regulatory networks of cells influencing non-response to LN drugs. **a**, Cell-cell communication networks that were found to specifically and significantly regulate subpopulations expressing non-response signatures to AZA and MMF, respectively. Imputed using CellChat R package. **b**, *In silico* imputed response rates for refractory patients to MMF and AZA using Hipathia R package. Response scores were estimated comparing transcriptome changes after and before gene-target inhibition obtained from the identified cell-cell regulatory networks.

As certain specific cell types express the non-response signatures to MMF and AZA, we aimed at identifying regulatory signaling across these cell subsets as potential therapeutic targets. We used CellChat R package^23^ to identify regulatory signaling networks between cell clusters specifically related with non-response to MMF and AZA followed by the analysis of their potential druggability using Hipathia R package^24^ (See Methods). Here, a theoretical response score was estimated for each patient from our cohort comparing changes at transcriptome level before and after inhibition of targets from each identified regulatory network. The CC-chemokine ligand (CCL) signaling network was found regulating the non-response signature to AZA, that is CD14+_cl4 and CD16 + monocytes (Fig. 5a). For clusters related with MMF non-response, the BAFF signaling network was identified as the best signaling route candidate (Fig. 5a). Interestingly, 63 percent of non-responder patients to AZA achieved a favorable estimated response by CCL inhibition against 40 percent for non-responders to MMF (Fig. 5b). BAFF inhibition reported favorable response for 74 and 56 percent of non-responders to MMF and AZA, respectively (Fig. 5b). In both cases, for MMF and AZA non-responders, response ratio was importantly increased to up 20 percent when inhibiting drug-specific non-response mechanisms. So, refractory patients for each drug could benefit from adding a tailored second therapy.

## Discussion

This study revealed different molecular and cellular mechanisms behind non-response to MMF and AZA by analyzing retrospectively a longitudinal cohort of responder and non-responder SLE patients to both drugs.

The course of the disease is complex and unpredictable, alternating periods of inactivity, disease flares and progression to organ damage, with different underlying molecular mechanisms which may potentially differ between patients. This heterogeneity particularly hinders the effective discovery of robust biomarkers for both disease progression as for treatment responses^17^. Cross-sectional studies of patients with active disease limit the different scenarios to analyze, reducing reproducibility in other cohorts and/or disease conditions. Therefore, a longitudinal cohort was selected, with samples representing different disease states, with different clinical manifestations and treated with different routine treatments and doses. Robust non-response gene signatures to MMF and AZA were obtained across all the clinical and molecular heterogeneity of the disease. Maintenance drugs including HC and HC plus GC were analyzed demonstrating that MMF and AZA non-response patterns were drug-specific, not influenced by secondary SOC therapies. In addition, drug signatures were used to build ML-based models to predict drug responses obtaining high performance results (balanced accuracies higher than 0.75 in all cases).

One main limitation of our study is the small number of patients treated using for some specific drugs (mainly for AZA), making more difficult the interpretation of the AZA-associated data. A larger interventional clinical trial would be required in order to validate responsiveness and non-responsiveness mechanisms to the drugs alone and to test the predictive capacity of the non-response signatures defined. In lupus, it is particularly difficult to obtain public longitudinal transcriptome data and more so if a single drug is to be studied. SLE patients take, in most instances, combinations of multiple drugs, and response outcomes are often not shared. Validation could bring us closer to more personalized medicine, supporting more effective first-line therapy choice for LN patients.

Despite this, we obtained revealing and encouraging results. Analyzing cell profiles, we observed a depletion of T cells in non-responder patients and a worse response ratio was consistently observed for patients poor in various T cell subpopulations. In a previous study, T lymphocyte exhaustion was associated with LN^25^, but differences comparing response and non-response to drugs have never been reported before. Perhaps insufficient or abnormal T cell function could be influencing the lack of response^26^. For MMF, the non-response was mainly mediated by PCs, pDCs and ABCs, in line with the fact that the worst response ratios were obtained for patients showing rich memory B cell profiles. ABCs are a class-switched, antigen-specific memory-like B cell population expanded in SLE that contributes to autoimmunity through the production of autoantibodies and cytokines and regulating inflammatory T cells acting as APCs^27^. Their differentiation is driven by the toll-like receptor (TLR) 7 in an interleukin-21-mediated mechanism^28^. Recently, expansion of ABCs has been observed in the kidneys of LN patients^29^ and in SLE mouse models^30^, underscoring the importance of these cells. The question remains as to why are ABCs remaining high and if this might be due to resistance of these cells to MMF, mechanisms that would need to be experimentally tested.

The MMF non-response signature was also expressed in NKT cells, which regulate Th1/Th2 balance^31^. In fact, cross-regulation between Tregs and NKT cells was previously reported. Activated NKT cells modulate Treg function through IL-2-dependent mechanisms, whereas Treg can suppress proliferation, cytokine release and cytotoxic activity of NKT cells by cell-contact-dependent mechanisms^32^.

CD1C + cDC2 and non-classical monocytes also over-expressed the non-response signature to MMF. cDC2 influence aberrant T cell functions secreting interleukin-8 and other proinflammatory cytokines^33^. HLA class II genes, expressed by APCs and importantly expressed by the relevant non-response-related cell subtypes, modulate the interaction of T and B cells in the production of autoantibodies. The genetic association of the HLA class II genes with autoantibody production in SLE is well established, and our results suggest that CD1C + cDC2 may be importantly involved in this context^34^. These clusters seem to be playing an important role in renal damage control, showing functions related to complement-mediated phagocytosis^22^. Complement cascade proteins bind immune-complex deposits in the kidney glomerulus driving immunopathology leading to long-time scars^35^.

For AZA, the most notable finding is the exacerbated expression of a non-response signature in CD16^+^ and CD14^+^ monocytes with genes involved in migration related functions. The accumulation of CD16^+^ monocytes in the blood could reflect either an increase in their differentiation, which would lead to greater amounts of them migrating to the target tissue, or just the opposite, a deficit in the correct migration processes to the tissue^36^. Deconvolution of cell types from bulk transcriptome did not allow identification of CD16^+^ monocytes in blood, so future analyses would be necessary to validate the increase or lack of migration of these monocytes to the tissue in the AZA therapy context.

Therefore, we revealed different molecular signatures and different cellular subtypes associated with them for non-response to MMF and AZA. In fact, *in silico* inhibition of targets from regulatory networks regulating clusters associated to MMF or AZA non-response identified different response ratios for refractory patients for each drug. CCL2 inhibition has been previously proposed to reduce tissue infiltration of monocytes, minimizing the inflammatory phenotypes^37^, while belimumab, an anti-BAFF drug, is currently approved for SLE and LN. BAFF inhibition leads to a reduction in autoantibody production, depleting the differentiation of PCs from B cells^38^. In fact, growing studies show the effectivity of combining belimumab with other immunosuppressant drugs^3^. Here, we presented potential evidence that anti-BAFF could be more beneficial for non-responders to MMF by *in silico* analysis. Detailed analysis is required to test the efficacy of belimumab as an add-on therapy to MMF in real world terms.

Finally, there is extensive evidence showing the importance of IFN-I in SLE and other autoimmune diseases^39,40^. We herein report the co-expression of IFN-related genes and non-response signatures to LN drugs in the same cell subsets. Specifically, at least a handful of genes from the *ISG* and *IFITM* families of genes showed high expression scores in subsets expressing AZA and MMF non-response signatures, both of them for AZA, and *IFITM* genes particularly for MMF.

The *IFITM*-family of genes codify 3 anti-viral subfamilies of proteins, one of which is immune-related, including, in turn, 3 main proteins, IFITM1, IFITM2 and IFITM3^41^, all of which evolved evolutionarily through their expansion and interaction with viral infections. Despite their protein sequence similarity IFITM1, 2 and 3 have different cellular localization and function, and different anti-viral specificity through mechanisms still poorly understood. While IFITM1 is exposed on the cell surface (former Leu-13 antigen-expressing cells, now CD225), IFITM2 and 3 are localized in endosomes and lysosomes. Interestingly, IFITM1 and IFITM3 have been found as part of the B cell signaling complex in the plasma membrane together with CD19 and CD21, as well as CD81. Upon B cell activation, IFITM3 protein is increased moving from the endosomes to the lipid rafts containing the B cell signaling complex. Most interestingly, several studies have addressed the role of IFITM3 in B cell activation with expansion and affinity maturation in germinal center B cells through amplification of the PI3K signaling pathway^41^. In B cell malignancies, expression of *IFITM3* is associated with poor outcomes^42^. In addition, IFITMs expression is induced by IFN-I primarily in monocyte-derived macrophages. Transcription is induced by various pro-inflammatory cytokines and Toll-like receptors agonists. The IFITM1–3 genes have an IFN response element that confers responsiveness to type I and II IFNs. So, IFITM and IFN-I regulate each other. What the function of these genes and others identified in non-responders is in the context of SLE, requires further investigation.

This new knowledge shed light on the molecular and cellular patterns associated to the non-response to LN therapies, opening a new scenario for further investigation of the regulatory mechanisms between implicated cell subsets, the genes and cells involved, and the development of new therapeutic strategies for LN and drug response prediction.

## Methods

### Study population

Lupus nephritis patients were recruited and followed for over 2 years at the Johns Hopkins University School of Medicine following the SPARE study protocol (Study of biological Pathways, disease Activity and Response markers in patients with systemic lupus Erythematosus)^43^. All patients gave written informed consent. Adult patients fulfilling the revised American College of Rheumatology classification criteria^44^ and ranging from 18 to 75 years were considered eligible. Patients were treated according to standard of care (OS and/or HC) and those treated with rituximab or other biologics at any visit were excluded. The doses were adjusted for each case according to the criteria of the physician. Starting from a retrospective analysis of 301 patients studied longitudinally and having gene expression data, we selected those who have been treated with MMF, AZA, HC or SOC, and with information for at least two visits since the start of treatment, allowing drug response follow-up. Samples treated with other immunosuppressant drugs in conjunction with MMF or AZA were discarded. These selection criteria led to the definite identification of 34, 11, 56 and 73 responder patients to MMF, AZA, HC and SOC, comprising 103, 24, 133 and 173 longitudinal samples, respectively, and 10, 9, 14 and 25 non-responsive patients to MMF, AZA, HC and SOC, comprising a total of 27, 30, 40 and 64 samples, respectively (Table 1). All selected patients showed historical abnormal findings in renal biopsies.

All clinical information was pseudo-anonymized. The medical history of the patients was collected including demographic information, medications used and autoantibody titers. To assess disease activity, the Safety of Estrogens in Lupus Erythematosus: National Assessment (SELENA) version of the Systemic Lupus Erythematosus Disease Activity Index (SLEDAI) and the Physician Global Assessment (PGA)^45^ were completed at each visit. Urinalysis, anti-dsDNA and plasma concentration of complement components 3 (C3) and 4 (C4) were also calculated at every visit. Response to drugs was defined using the SRI-4^46^ considering at least at 3 months from the first visit with the specific drug, but only patients who maintained the response over time while the drug was being used were considered responders (16.25 months on average). For MMF and AZA, a second response outcome was defined over time according to whether the protein/creatinine ratio in urine was reduced and maintained below 500 mg/g from at least 3 months until the last visit under treatment.

### Data preparation

Peripheral blood samples were collected at each visit using the PAXgene blood RNA system and gene expression profiles were measured using Affymetrix GeneChip HT HG-U133 + arrays. The experimental protocol from data preparation to gene expression data preprocessing has been previously reported^43^. Expression values were transformed to logarithmic scale and transcripts were annotated from probes to official gene nomenclature (Gene Symbol). Duplicated genes were merged assigning their mean expression value and genes with flat expression profiles were filtered out.

### Differential expression and functional analysis

Transcriptome analysis was used to identify the genes and molecular mechanisms behind drug response and non-response to each therapy. First, clinical and demographic confounders were identified using the swamp R package^47^. Samples from the same patient, doses of MMF and prednisone, disease activity, race, and sex were the variables that explained the greatest variance in the data, in decreasing order. DEGs obtained comparing response and non-response were analyzed by linear mixed models using the limma R package^48^ adjusting expression values for sex, patient, SLEDAI, prednisone and MMF or AZA dose. Thus, we obtained genes with significant differential expression between responders and non-responders, independently of treatment and doses used, sex, and conserved longitudinally across different visits, different disease states and disease activity fluctuations. Genes with a Bonferroni-corrected p-value < 0.05 were considered significant. Data were not adjusted for race because a significant imbalance in the distribution of race between both groups of patients was observed for some therapies (Table 1).

The functional role of DEGs was investigated using qusage R package^49^ using a set of blood immune-related gene-modules previously described^50,51^.

### Machine learning-based predictive models

Differential gene expression signatures from longitudinally sampled SLE patients were used as features to build ML-based models to predict responses to MMF, AZA, HC and SOC, independently. In detail, nested k-fold cross validation was implemented^52^ (Supplementary Fig. 4a). First, the entire dataset was divided into 5 class-balanced folds selecting 80 and 20% of the samples as training and test sets. Samples from the same patient were forcibly assigned to the same group (train or test). Hyperparameters for models were tuned by inner 10-fold cross-validation for each training set, repeated 5 times with internal random initialization, where 90 and 10% of the samples were assigned to internal train and test sets. A total of 11 different classification algorithms were tested including gaussian linear model, linear discriminant analysis, extreme gradient boosting, random forest, k-nearest neighbors, linear and radial super vector machine, neural networks, naive Bayes, boosted classification trees and boosted generalized additive model, covering the main ML approaches^53^. Model performances were calculated in each separated outer test fold and the algorithm prioritization was based on the average of MCC values obtained across outer folds to give an unbiased measurement of model accuracy. R code used to build ML-based models was available at https://github.com/jordimartorell/pathMED.

### Cell profiling

Blood cell subtype proportions were deconvoluted from gene expression data using CibersortX^54^. A reference panel with markers for 22 different cell types were downloaded from the Cibersort website. Macrophage and mastocyte proportions were discarded as they are not blood circulating populations. Following deconvolution, patients were labeled as rich/poor for each individual cell type based on the median value of the cell type across all patients (rich or poor if the cell proportion is higher or lower than the median proportion, respectively)^55^.

### Single-cell analysis

Raw single-cell RNA-seq data from peripheral blood mononuclear cells for 41 SLE patients was downloaded from The National Center for Biotechnology Information Gene Expression Omnibus (NCBI GEO) database^56^ (ID: GSE135779)^39^. All the analyses were carried out with R mainly using the Seurat package^21^. Cells with percentage of mitochondrial counts > 25%, percentage of ribosomal counts > 25%, number of unique features or total counts outside 0.5–99.5% range of all cells, number of unique features < 200 or Gini or Simpson diversity index < 0.8, were discarded. In addition, mitochondrial and ribosomal genes and genes expressed in fewer than 5 cells were removed. Doublets were also removed using scDblFinder R package^57^. Total counts per cell were normalized and fixed to 1000 and gene counts were log transformed. Feature values were standardized by mean centering and standard deviation scaling and then, values per cell were adjusted correcting by cell cycle scoring and mitochondrial counts. Finally, data integration across cells was performed using Harmony^58^. Louvain algorithm and Uniform Manifold Approximation and Projection (UMAP)^59^ were used to cluster and to visualize the clusters of cells. Cluster stabilities were measured using the clustree R package^60^.

Cells were annotated with major blood cell type labels by correlation with cell markers previously defined by Nehar-Belaid and colleagues^39^. To identify specific cell subtypes or subclusters within each major cell type, the entire process was carried out from the start excluding remaining cells not cataloged with that particular major cell type. In this way, adequate resolution is reached to cluster minor cell types. Gene-markers for each subcluster were obtained comparing each subcluster with the rest of clusters within the same cell type using the FindMarkers function from Seurat. Cell tagging was performed using published cell-marker annotations^61–63^. The average expression levels for gene-signatures (expression score) for each cluster were calculated using AddModuleScore function from Seurat R package, subtracted by the aggregated expression of randomly selected control gene sets, to identify the specific cell clusters in which a certain gene-signature was particularly represented or was more expressed.

### Statistical analysis

The Wilcoxon Mann-Whitney and Fisher’s exact tests were used to identify significant associations between response/non-response in continuous and categorical clinical variables, respectively. Demographic variables and medical history were analyzed at patient level and variables that change over time, as the SLEDAI or the serum component levels, were analyzed by sample considering all visits.

Regarding cells, the Wilcoxon-Mann Whitney test was also used to define the significance when comparing cell proportions between responder and non-responder patients. Significant differences in response rates (percentage of responder samples/total of samples) comparing two groups of patients were obtained by Fisher’s exact test.

GSEA was used to compare the similarity between DEG lists obtained for each drug^20^. A similarity score was obtained for each pair of drugs according to if DEGs for a drug were randomly distributed, at the top (positive score) or at the bottom (negative score) throughout the sorted gene list (by fold change, comparing responder and non-responder samples) of the second drug.

### Inference of druggability through targets-inhibition

Intercellular communication networks were inferred from single-cell data using CellChat^23^ and major signaling input and output processes between previously defined cell clusters were revealed. Then, we focused on signaling networks that specifically regulated non-response-related subclusters as potentially druggable networks. Targets for each druggable network were extracted from CellChat internal database (list of genes from each signaling network). We used Hipathia R package^24^ to estimate the effect of target inhibitions on gene expression on patients from our cohort, following the instructions provided by the authors. A response score for the inhibition of targets from each druggable network was calculated for each patient as the absolute change of gene expression before and after the target inhibition. The expression of the targets was multiplied by 0.1 to simulate inhibition (http://hipathia.babelomics.org), and expression changes on the whole transcriptome were imputed using mechanistic models based on biology-based knowledge. Anticipated favorable response to inhibition of a specific druggable network in a patient was defined as a response score equal to or greater than the mean response scores of all patients. Percentage of patients having favorable response score was calculated from the total of non-responder patients to MMF and AZA independently.

## Figures and Tables

**Table 1:** Characteristics of the patients included in the study.

	MMF	AZA	HC	SOC

	Responder/Non-responder	Responder/Non-responder	Responder/Non-responder	Responder/Non-responder

Patient (samples)	34 (103) / 10 (27)	11 (24) / 9 (30)	56 (133) / 14 (40)	73 (173) / 25 (64)

Demographic				

Female sex	28 (82.3%) / 9 (90%)	9 (81.8%) 9 (100%)	53 (94.6%) 12 (85.7%)	70 (95.9%) / 21 (80%)

**Race**	**C: 22 (64.7%) / 2 (20%)** *	C: 6 (54.5%) / 3 (33.3%)	C: 19 (33.9%) / 4 (28.6%)	C: 26 (35.6%) / 8 (32%)
	**AA: 7 (20.59%) / 7 (70%)** **	AA: 4 (36.4%) / 5 (55.6%)	AA: 34 (60.7%) / 9 (64.3%)	AA: 44 (60.3%) / 15 (60%)
	O: 5 (14.7%) / 1 (10%)	O: 1 (9.1%) / 1 (11.1%)	O: 3 (5.4%) / 1 (7.1%)	O: 3 (4.1%) / 2 (8%)

Age	27.78 (± 11.5) / 25.1 (± 10.3)	29.26 (± 12.1) / 26.38 (± 8.9)	34.2 (± 13.2) / 28 (± 11.2)	32.79 (± 13.1) / 26.64 (± 9.7)

Weight	171.17 (± 54.7) / 179.67 (± 47.4)	174.29 (± 44.3) / 171 (± 42.2)	173.92 (± 42.3) / 173.9 (± 51.4)	170.44 (± 41.4) / 170.33 (± 46.8)

Family history of SLE	11 (32.25%) / 4 (40%)	2 (18.18%) / 2 (22.22%)	18 (32.14%) / 6 (42.85%)	26 (35.62%) / 10 (40%)

**SLEDAI**	**2.94 (± 3.2) / 4.44 (± 3.3)** *	**1 (± 1.3) / 4.53 (± 2.2)** ****	**0.96 (± 2.8) / 2.67 (± 2.4)** ****	**1.13 (± 1.9) / 3 (± 2.9)** ****

Treatments (mg)				

**MMF**	**1551.94 (± 900.6) / 2444.44 (± 974)** ****	0 (± 0) / 0 (± 0)	0 (± 0) / 0 (± 0)	0 (± 0) / 0 (± 0)

AZA	0 (± 0) / 0 (± 0)	147.92 (± 39.6) / 191.38 (± 256.7)	0 (± 0) / 0 (± 0)	0 (± 0) / 0 (± 0)

**Prednisone**	**4.98 (± 7.2) / 10.37 (± 13.5)** *	3.33 (± 3.8) / 5.2 (± 6.7)	0 (± 0) / 0 (± 0)	**1.32 (± 3.2) / 2.57 (± 4.1)** **

**Asa**	**33.68 (± 43.3) / 56.08 (± 38.1)** *	50.67 (± 75) / 33.52 (± 40.6)	35.91 (± 64.8) / 34.42 (± 40.6)	38.38 (± 65.5) / 36.7 (± 40.6)

Plaquenil	79 (76.7%) / 20 (74.1%)	20 (83.3%) / 25 (83.3%)	130 (97.7%) / 40 (100%)	170 (98.3%) / 63 (98.4%)

**NSAID**	5 (4.9%) / 3 (11.1%)	2 (8.3%) / 2 (6.7%)	**46 (34.6%) / 3 (7.5%)** ***	**56 (32.4%) / 10 (15.6%)** ***

Antihypertensive	72 (69.9%) / 21 (77.8%)	16 (66.7%) / 20 (66.7%)	78 (58.6%) / 22 (55%)	99 (57.2%) / 41 (64.1%)

Diuretic	28 (27.2%) / 9 (33.3%)	7 (29.2%) / 7 (23.3%)	41 (30.8%) / 13 (32.5%)	58 (33.5%) / 21 (32.8%)

Calcium channel	13 (12.6%) / 2 (7.4%)	7 (29.2%) / 7 (23.3%)	18 (13.5%) / 4 (10%)	20 (11.6%) / 11 (17.2%)

ACE inhibitor	66 (64.1%) / 20 (74.1%)	14 (58.3%) / 18 (60%)	60 (45.1%) / 15 (37.5%)	73 (42.2%) / 32 (50%)

Statin	37 (35.9%) / 6 (22.2%)	5 (20.8%) / 8 (26.7%)	23 (17.3%) / 3 (7.5%)	29 (16.8%) / 12 (18.8%)

Serological				

Anti-dsDNA (+)	28 (82.4%) / 8 (80%)	7 (63.6%) / 8 (88.9%)	22 (39.3%) / 11 (78.6%)	31 (42.5%) / 18 (72%)

**Anti-Sm (+)**	15 (44.1 %) / 3 (30%)	2 (18.2%) / 2 (22.2%)	3 (5.4%) / 3 (21.4%)	**4 (5.5%) / 8 (32%)** **

Anti-ANA (+)	32 (94.1%) / 10 (100%)	10 (90.9%) / 9 (100%)	73 (100%) / 25 (100%)	73 (100%) / 25 (100%)

Anti-Ro (+)	13 (38.2%) / 4 (40%)	5 (45.5%) / 3 (33.3%)	17 (30.4%) / 5 (35.7%)	21 (28.8%) / 9 (36%)

Anti-La (+)	7 (20.6%) / 2 (20%)	1 (9.1%) / 2 (22.2%)	9 (16.1%) / 2 (14.3%)	10 (13.7%) / 3 (12%)

Anti-RNP (+)	16 (47.1 %) / 5 (50%)	1 (9.1%) / 4 (44.4%)	9 (16.1%) / 3 (21.4%)	11 (15.1%) / 8 (32%)

Anti-B2Gly (+)	12 (35.3%) / 3 (30%)	2 (18.2%) / 4 (44.4%)	13 (23.2%) / 5 (35.7%)	17 (23.3%) / 9 (36%)

C3 level* (mg/dl)	**103.19 (± 33.7) / 133.96 (± 26.4)** ****	**109.42 (± 25.8) / 80.93 (± 31.9)** ***	**131.13 (± 32.1) / 102.95 (± 39.2)** ****	**129.74 (± 32.8) / 107.97 (± 37.8)** ****

C4 level* (mg/dl)	**18.33 (± 8.5) / 25.32 (± 5)** ****	**22.7 (± 7) / 16.69 (± 9.2)** ***	**25.04 (± 9.1) / 18.6 (± 8)** ****	**24.74 (± 9.1) / 20.98 (± 11)** ****

**Anti-DNA titers**	**57.73 (± 135.6) / 5.36 (± 19.1)** ***	**5.04 (± 11) / 262.07 (± 239.9)** ****	**8 (± 37.7) / 49.8 (± 129.3)** ***	**7.58 (± 35.3) / 33.16 (± 104.2)** ***

**IgG ACL titers**	11.82 (± 17.7) / 8.32 (± 6.3)	8.13 (± 7.1) / 8.57 (± 3.3)	**6.94 (± 4.3) / 12.77 (± 21.1)** *	6.86 (± 4) / 10.08 (± 17.2)

**IgM ACL titers**	7.21 (± 3.9) / 7.88 (± 3.8)	7.91 (± 4) / 7.53 (± 3)	**8.54 (± 10.2) / 13.1 (± 20.9)** *	**8.58 (± 9.3) / 11.79 (± 17)** *

**IgA ACL titers**	**4.2 (± 3.1) / 5.44 (± 3.2)** *	4.17 (± 2.7) / 4.13 (± 2.7)	**3.67 (± 3.7) / 6.21 (± 6.5)** **	**3.63 (± 3.4) / 5.39 (± 5.6)** *

Data is presented as the number of patients or samples (and percentage) for categorical variables or means (± standard deviation) for numerical variables. P-values were calculated using the Wilcoxon-Mann Whitney test and Fisher’s exact test for quantitative and categorical measurements, respectively. P-values < = 0.05 were assessed as significant and marked with asterisks based on significance magnitude

* = <0.05

** = <0.005

*** = <0.0005

**** means p-value lower than 0.00005.

Treatments used, SLEDAI, C3 and C4 levels and antibody titers were analyzed by sample considering all visits, while demographic information and autoantibody positivity (+) were analyzed at individual patient level. C: Caucasian; AA: African American; O: Other; Asa: acetylsalicylic acid; NSAID: non-steroid anti-inflammatory drugs; ACE: angiotensin-converting-enzyme; B2Gly: b2 glycoprotein; ACL: anticardiolipin; ANA: antinuclear antibody; anti-RNP: antinuclear ribonucleoprotein.

## Data Availability

Expression data generated from all samples from patients described in the manuscript are available in NCBI GEO database^56^, under identifier code: GSE224705. Drug response information, doses, demographic information and visits-patient identification are also included. All published data were anonymized.
